# Preparation and Characterization of Solid Dispersions of Artemether by Freeze-Dried Method

**DOI:** 10.1155/2015/109563

**Published:** 2015-05-17

**Authors:** Muhammad Tayyab Ansari, Altaf Hussain, Sumaira Nadeem, Humaira Majeed, Syed Saeed-Ul-Hassan, Imran Tariq, Qaisar Mahmood, Abida Kalsoom Khan, Ghulam Murtaza

**Affiliations:** ^1^Faculty of Pharmacy, Bahauddin Zakariya University, Multan 6000, Pakistan; ^2^College of Pharmacy, University of the Punjab, Lahore 54000, Pakistan; ^3^Department of Environmental Sciences, COMSATS Institute of Information Technology, Abbottabad 22060, Pakistan; ^4^Department of Chemistry, COMSATS Institute of Information Technology, Abbottabad 22060, Pakistan; ^5^Department of Pharmacy, COMSATS Institute of Information Technology, Abbottabad 22060, Pakistan

## Abstract

Solid dispersions of artemether and polyethylene glycol 6000 (PEG6000) were prepared in ratio 12 : 88 (group-1). Self-emulsified solid dispersions of artemether were prepared by using polyethylene glycol 6000, Cremophor-A25, olive oil, Transcutol, and hydroxypropyl methylcellulose (HPMC) in ratio 12 : 75 : 5 : 4 : 2 : 2, respectively (group-2). In third group, only Cremophor-A25 was replaced with Poloxamer 188 compared to group-2. The solid dispersions and self-emulsified solid dispersions were prepared by physical and freeze dried methods, respectively. All samples were characterized by X-ray diffraction, attenuated total reflectance Fourier transform infrared spectroscopy, differential scanning calorimeter, scanning electron microscopy, and solubility, dissolution, and stability studies. X-ray diffraction pattern revealed artemether complete crystalline, whereas physical mixture and freeze-dried mixture of all three groups showed reduced peak intensities. In attenuated total reflectance Fourier transform infrared spectroscopy spectra, C–H stretching vibrations of artemether were masked in all prepared samples, while C–H stretching vibrations were representative of polyethylene glycol 6000, Cremophor-A25, and Poloxamer 188. Differential scanning calorimetry showed decreased melting endotherm and increased enthalpy change (Δ*H*) in both physical mixture and freeze-dried mixtures of all groups. Scanning electron microscopy of freeze-dried mixtures of all samples showed glassy appearance, size reduction, and embedment, while their physical mixture showed size reduction and embedment of artemether by excipients. In group-1, solubility was improved up to 15 times, whereas group-2 showed up to 121 times increase but, in group-3, when Poloxamer 188 was used instead of Cremophor-A25, solubility of freeze-dried mixtures was increased up to 135 times. In fasted state simulated gastric fluid at pH 1.6, the dissolution of physical mixture was increased up to 12 times and freeze-dried mixtures up to 15 times. The stability of artemether was substantially enhanced in freeze-dried mixtures by using polyethylene glycol 6000, Cremophor-A25, and Poloxamer 188 of self-emulsified solid dispersions of artemether in Hank's balanced salt solution at pH 7.4.

## 1. Introduction

Malaria is the infection caused by protozoan parasites transmitted with female Anopheles mosquitoes belonging to the genus,* Plasmodium* [1]. The five different species of* Plasmodium* which are the basis of malarial disease in many human beings are* P. vivax, P. falciparum, P. malariae, P. ovale, *and* P. knowlesi*. The symptoms of malaria at first may be nonspecific such as joint pain, abdominal pain, asthenia, high fever, anorexia, and vomiting as well as shivering.* P. falciparum* may cause the most severe form of malaria particularly in children as well as in nonimmune travellers who are from nonendemic countries and also in pregnant women [2].

According to a wide survey of malaria, ninety-nine countries out of 106 malaria endemic countries had ongoing malaria transmission. About 3.3 billion people in the world were endangered of malaria according to estimation. Malaria is the infectious disease as well as most prevalent disease in the world which in each year affects 515–600 million humans. About 40% of world population was vulnerable to malarial infection. In 2010, an estimated 655,000 persons died because of malaria; out of them, 86% were only the children with age of less than five years [3].

In order to advance the cure rates and clinical responses as well as to slow the development of resistance of malaria parasite, WHO has suggested that artemisinin derivatives should be present in antimalarial regimens. Artemether (ARTM) which is artemisinin derivative reduces malaria transmission and may also reduce the gametocyte carriage [4, 5]. ARTM belongs to artemisinin family and is the active component of the Chinese herbs, that is, the* qinghao*, known as* Artemisia annua*. ARTM has quick start of the schizontocidal actions and then it is metabolized in the liver into its demethylated derivatives, known as dihydroartemisinin (DHA). ARTM has been proved to be efficient against acute* P. falciparum* as well as uncomplicated malaria. Its structural unit consists of 1,2,4-trioxane ring constituting the active pharmacophore of the ARTM which was responsible for its antimalarial activity [6]. Solubility of ARTM in water is poor, so it has been synthesized in tablets as well as intramuscular injections, but short half-life of about 3 to 5 hours is the significant drawback of ARTM [7]. ARTM results in incomplete absorption after oral administration due to poor aqueous solubility. By using more water soluble formulation, the solubility and dissolution can be increased [8].

**Figure 1:**
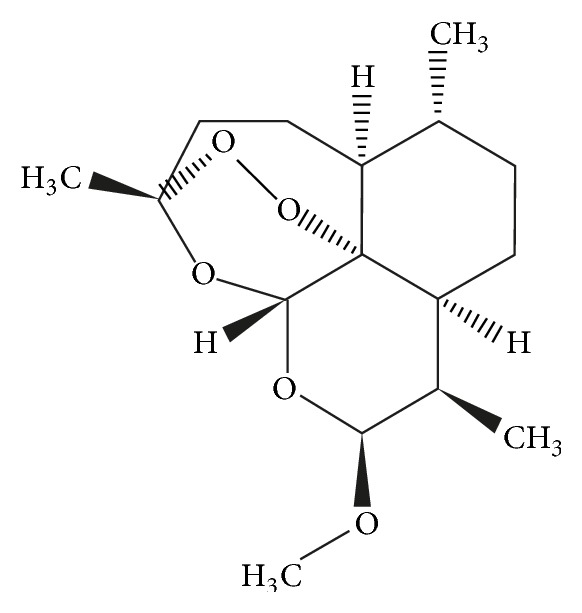
Structure of the ARTM [10].

In the literature, various technological strategies are reported such as solid dispersions, self-emulsifying drug delivery systems (SEDDSs), micronizations, and complex formation with cyclodextrins [9]. The chemical structure of ARTM is shown in Figure 1.

The aim of this study was to prepare self-emulsified solid dispersions (SESDs) of ARTM by using PEG6000, Poloxamer 188, Cremophor-A25, Transcutol, olive oil, and HPMC in order to improve solubility and dissolution behavior of ARTM.

## 2. Materials and Methods

### 2.1. Materials

Artemether (ARTM) (Alchem, China), acetonitrile HPLC grade (Merck, Germany), analytical grade methanol (Merck, Germany), Cremophor-A25 (chemically known as polyethylene glycol 1100 mono(hexadecyl/octadecyl) ether, YunGou Chemicals, China), polyethylene glycol 6000 (PEG 6000, Fluka, USA), Poloxamer 188 (chemically known as poly(ethylene glycol)-*block*-poly(propylene glycol)-*block*-poly(ethylene glycol), YunGou Chemicals, China), olive oil (Mezoa Chemicals, Spain), hydroxypropyl methylcellulose-K15M (HPMC-K15M, Fluka, USA), Transcutol (chemically known as 2-(2-ethoxyethoxy)ethanol, YunGou Chemicals, China), starch (Fluka limited company), lactose (as monohydrate, DMV International, Pakistan), Primogel (chemically known as sodium Starch glycolate, Yung Zip Chemicals, China), magnesium stearates (Mg Stearate, Royal Tiger, Pakistan), potassium bromides (KBr, Merck, Germany), hydrochloric acid (HCl, Merck, Germany), sodium chloride (NaCl, Sigma-Aldrich, Germany), sodium taurocholate (Sigma-Aldrich, Germany), and silica gel were purchased through commercial sources and used without further treatment.

### 2.2. Physical Mixture (PM) Method

Physical mixtures (PMs) were prepared using weighed amount of ARTM and PEG6000 in ratio 12 : 88 named group-1 and ARTM, PEG6000, Cremophor-A25, olive oil, Transcutol, and HPMC with ratio 12 : 75 : 5 : 4 : 2 : 2, respectively, named group-2. Similarly, ARTM, PEG6000, Poloxamer 188, olive oil, Transcutol, and HPMC with ratio 12 : 75 : 5 : 4 : 2 : 2, respectively, was named group-3. These physical mixtures were dried in oven at 37°C and then, after complete drying, homogenous mixture was made by using pestle and mortar with soft grinding. These mixtures were passed through a sieve of 180 *μ*m mesh size, placed in dried, labeled brown glass bottles and then kept in desiccators at room temperature for further analysis.

### 2.3. Freeze-Dried (FD) Method

Via freeze drying method, soluble mixtures of weighed amount of ARTM and PEG6000 in ratio 12 : 88 (Group-1) and ARTM, PEG6000, Cremophor-A25, olive oil, Transcutol, and HPMC with ratio 12 : 75 : 5 : 4 : 2 : 2, respectively (group-2) were prepared. Similarly ARTM, PEG6000, Poloxamer 188, olive oil, Transcutol and HPMC with ratio 12 : 75 : 5 : 4 : 2 : 2, respectively (group-3) were mixed to prepare soluble mixture. According to these corresponding groups, solutions were transferred to round bottom flasks and shaked on orbit shaker (BioTechnics, India) for mixing. After proper mixing, solvents were evaporated by using rotary evaporator (Prolific Instruments, India). Then, small amount of deionize water was added, shaken well, and frozen at temperature of −70°C to −80°C in the electronic deep-freezer (Dawlance, Pakistan). The frozen form is then freeze-dried using lyophilizer (Labconco, England) at temperature of −42°C using vacuum of 0.100 mBar for complete removal of solvents. After complete drying, the freeze-dried (FD) mixtures were transferred to pestle and mortar, softly grinded, and passed through a sieve (180 *μ*m). These preparations were then stored in dried, labeled brown glass bottles and stored in desiccators for further process.

### 2.4. X-Ray Diffraction Studies

The X-ray powder diffraction (XRD) study of all samples was done by using apparatus named Siemens D-500. The measurements and conditions of XRD consisted of the targeting of CuK*α*, by using voltage of 40 KV and the current of 30 mA. A modified system of diverging and receiving as well as receiving and antiscattering slits of 1°, 1°, 1°, and 0.15°, respectively, was utilized. For data processing, Jade 6.0 (Materials Delta Inc.) was used. By utilizing a step width of about 0.04° 2*θ* between 5° and 50°, the XRD patterns were obtained.

### 2.5. Attenuated Total Reflectance Fourier Transform Infrared (ATR-FTIR) Spectrophotometric Analysis

By using potassium bromide (KBr) disc method (i.e., 0.5–1% of the sample in 200 mg KBr disc), ATR-FTIR spectra of SESDs of ARTM were obtained through Perkin Elmer spectrum 1. The scanning was at 400–4000 cm^−1^ and a resolution was then 1 cm^−1^. Instrument calibration was occasionally repeated during these operations.

### 2.6. Differential Scanning Calorimetric Analysis

Differential scanning calorimetric (DSC) analysis of physical and freeze-dried mixtures of ARTM and excipients was performed by using Q2000 DSC (TA instrument, USA). The samples were heated at a rate of 5°C/min from 25 to 250°C under a dry nitrogen gas purge. Tzero aluminum was used to calibrate the cell constant. All measurements were conducted in sealed nonhermetic aluminum pans. The typical sample weight was 5–10 mg.

### 2.7. Scanning Electron Microscopy

In order to identify and confirm the nature as well as surface topography of all formulated samples of ARTM, scanning electron microscopy (SEM, Perkin Elmer, USA) was used. SEM analysis was also performed to study the morphologies of pure drug as well as different self-emulsifying agents. For scanning electron photographs, an accelerating voltage of 5 kV was utilized and the resultant micrographs were then examined at magnifications of ×1000, ×1500, and ×2500.

### 2.8. Equilibrium Solubility Studies

For solubility in equilibrium studies, 0.4 g of each group was weighed properly and then transferred into test tubes containing 10 mL of the deionized water and mixed by using vortex mixture for period of about 1 to 2 min at 1400 revolutions per minutes (RPM). The prepared samples were fixed on orbit shaker for mixing and shaken for a period of 7 days at about 150 RPM at a temperature of 37°C. After a period of 7 days, each sample was then centrifuged at about 6000 RPM for 20 min. Then, upper layer of about 5 mL was decanted carefully by using micropipette and was then further diluted with 20 mL of deionized water. They were then analyzed on HPLC at 215 nm ultraviolet (UV) wavelength.

### 2.9. Preparation and Characterization of Tablets

Tablets were prepared employing direct compression method using single punch tablet machine. To make tablets, the homogenous mixture of preformulated grains and lactose (quantity sufficient for 500 mg tablet weight) was prepared followed by passing through a sieve of 180 *μ*m mesh size. Magnesium stearate (0.5%), Primogel (5%), and talcum powder (0.5%) were also mixed with these grains and mixing was carried out for about 10–20 min. The weight of each tablet was 500 mg, out of which 333 mg consisted of granules containing 40 mg of pure ARTM and another portion was 167 mg consisting of inactive material. The prepared tablets of different formulations were then stored and labeled properly. For assessment of quality, these tablets were characterized for various compendial requirements including weight variation, friability, and drug contents.

### 2.10. Dissolution Studies

The dissolution studies of all formulations of ARTM were done by utilizing USP dissolution apparatus II (Digitek, Lahore, Pakistan) with stirring speed of 100 RPM at 37°C. Fasted state simulated gastric fluid (FaSSGF) with pH of 1.6 with composition of sodium taurocholate 80 *μ*M, sodium chloride (NaCl) 34.2 mM, hydrochloric acid (HCl) q.s. to adjust pH to 1.6, and deionized water q.s. to make 1 L with pH 1.6 was used as biorelevant dissolution medium. The tablets containing SESDs of ARTM as well as other excipients in various ratios were put in dissolution medium of about 1000 mL. In the dissolution experiment, each tablet contained a specific quantity of powder in which 40 mg ARTM was present. On specific time intervals such as 5, 15, 30, 60, 90, 120, 180, and 240 min, aliquots of about 10 mL were taken out which were replaced through the addition of 10 mL of fresh FaSSGF. These obtained samples were then analyzed using HPLC at 215 nm. The obtained dissolution data was analyzed using various kinetic models including zero order, first order, Higuchi, and Korsemeyer-Peppas model. Indifferent to other models, Korsmeyer-Peppas model involves the fitting of initial 60% drug release data to find out the mode of drug release, *n* [8].

### 2.11. Stability Studies

For pure ARTM, SDs, and SESDs of ARTM, the stability tests in Hank's balanced salt solutions were carried out at 37°C that indicated the dissolution test temperature. The Hank's balanced salt solution was formulated with 0.40 gL^−1^ KCl, 8.00 gL^−1^ NaCl, 0.06 gL^−1^ KH_2_PO_4_, 0.35 gL^−1^ NaHCO_3_, 0.19 gL^−1^ CaCl_2_·2H_2_O, 0.05 gL^−1^ Na_2_HPO_4_, 0.09 gL^−1^ MgSO_4_, and 1.00 gL^−1^ glucose and the pH was adjusted to 7.4 (with NaHCO_3_, 3.8 mM, pH 11.2, solution). For stability tests in Hank's balanced salt solutions (pH 7.4), ARTM and its SESDs solution with concentration of 100 *μ*g mL^−1^ were firstly put into 10 mL test tubes with plugs. Then, aliquots of about 0.5 mL were taken out at intervals of 1 hour at 37°C in 6 hour. The concentration of pure ARTM and prepared samples was measured by HPLC at 215 nm and each test was performed in triplicate. Since the degradation of ARTM followed first-order kinetics, apparent degradation rate constants (*k*) were used to calculate the stability of ARTM and its SESDs from the slope of the degradation diagrams according to the following equation:

(1) $$\begin{array}{c} \ln\left[C\right]=\ln\left[C_{o}\right]-kt,\;\left(C\neq C_{o}\right), \end{array}$$

where [*C*_*o*_] was the initial concentration of ARTM, [*C*] was the concentration of ARTM at time *t*, and *k* was the slope of the fitted linear regression for the first order reaction.

### 2.12. High Performance Liquid Chromatography (HPLC) Analysis

The supernatant solutions of each group of SESDs of ARTM were withdrawn and then filtered through the cellulose acetates filters of 0.22 *μ*m in pore size. The amount of drug dissolved was then analyzed by using HPLC (Perkin Elmer, USA) at 215 nm after suitable dilution. This assay was determined by using reverse phase C18 column (4.6 mm × 250 mm, 5 *μ*), while UV detector was set at a wavelength of about 215 nm. Mobile phase consisted of a mixture of acetonitrile and water (75 : 25, v/v) operated at a flow rate of 1 mL/min. The injection volume was 20 *μ*L.

The validation data shows that the used HPLC method follows linearity in the range of 0.078 to 2.5 mg, as evident from the value of *R* ^2^ = 0.999 with *Y* = 462.5*X* − 21.32. It relates to the closeness of the test results to true values, that is, measure of exactness of analytical method. It is expressed as percentage recovery by the assay of known amount of analyte in the linearity range. For the determination of accuracy, the ARTM percentage recovery was 99.98, 100.34, 101.64, 101.75, 101.83, and 101.94% for dilutions 0.078, 0.1562, 0.3125, 0.625, 1.25, and 2.5 mg/mL, respectively. The accuracy and precision of method were 99.31 ± 2.94 and 98.72 ± 2.02, respectively [11].

### 2.13. Statistics

In all cases, analysis of the data was carried out by applying one-way ANOVA with a probability of *P* < 0.05 set as statistically significant.

## 3. Results and Discussion

### 3.1. X-Ray Diffraction Studies

**Figure 2:**
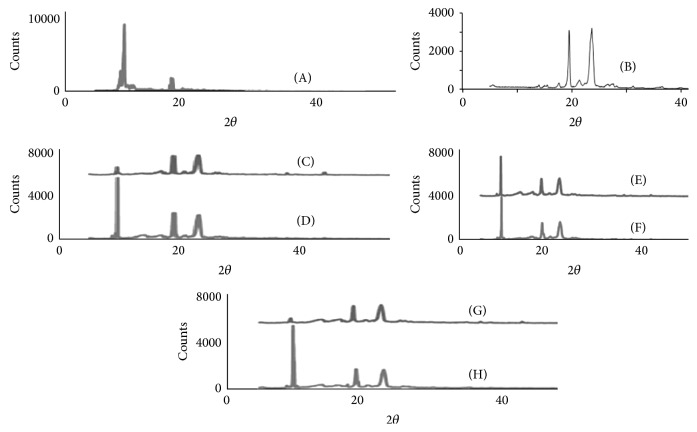
X-ray diffraction patterns of ARTM (A), PEG6000 (B), physical mixture of group-1 (C), freeze-dried mixture of group-1 (D), physical mixture of group-2 (E), freeze-dried mixture of group-2 (F), physical mixture of group-3 (G), and freeze-dried mixture of group-3 (H).

The XRD patterns of artemether (ARTM) showed very strong characteristic diffraction peaks at 2*θ* of 9.88°, 17.64°, 18.04°, and 19.68°. It signifies that artemether is purely a crystalline compound (Figure 2(A)). The XRD pattern of PEG6000 showed characteristic diffraction peaks at 2*θ* of 19.6° and 23.76° (Figure 2(B)).

Poloxamer 188 is crystalline in nature and gives three characteristic peaks, that is, at 19°, 22°, and 23° [11]. X-ray diffraction analysis of physical mixture of group-1 showed characteristic diffraction peaks at 2*θ* of 9.80°, 19.20°, and 23.40°, similarly freeze-dried mixture of group-1 showed diffraction peaks at 2*θ* of 9.72°, 19.12°, and 23.32°. These peaks represent ARTM and PEG6000. It was noted that intensity of ARTM diffraction peaks in physical mixture of group-1 was lower than the intensity of pure ARTM, while in freeze-dried mixtures crystalline peaks of ARTM were very less intense than pure ARTM (Figures 2(C) and 2(D)).

The X-ray diffraction analysis of physical mixture of group-2 showed characteristic diffraction peaks at 2*θ* of 9.88°, 19.28°, and 23.44°, while its freeze-dried form showed peaks at 2*θ* of 9.72°, 19.12°, and 23.36°, respectively. These peaks represent ARTM and PEG6000 also. The principal ARTM peak of ARTM and PEG6000 in physical and freeze-dried mixtures of group-2 were present but having lower intensity compared to pure ARTM and this decrease of intensity were more pronounced in freeze-dried mixture than in physical mixture (Figures 2(E) and 2(F)), as also seen for rofecoxib [12].

When Poloxamer 188 was incorporated in place of Cremophor-A25 in SESDs compared to XRD of physical mixture of group-3, SESDs showed diffraction peaks at 2*θ* of 10.12°, 19.48°, and 23.76° and its freeze-dried mixture showed peaks at 2*θ* of 9.64°, 19.04°, and 23.20°, respectively, which were representative of ARTM and PEG6000. The peak intensity of two peaks of PEG6000 in both physical and freeze-dried mixture of group-3 was substantially decreased, while principal ARTM peak in its freeze-dried mixture was 12 times less than intense compared to pure ARTM (Figures 2(G) and 2(H)), as also seen for rofecoxib [12, 13].

### 3.2. Attenuated Total Reflectance Fourier Transform Infrared Spectroscopy (ATR-FTIR) Studies

**Figure 3:**
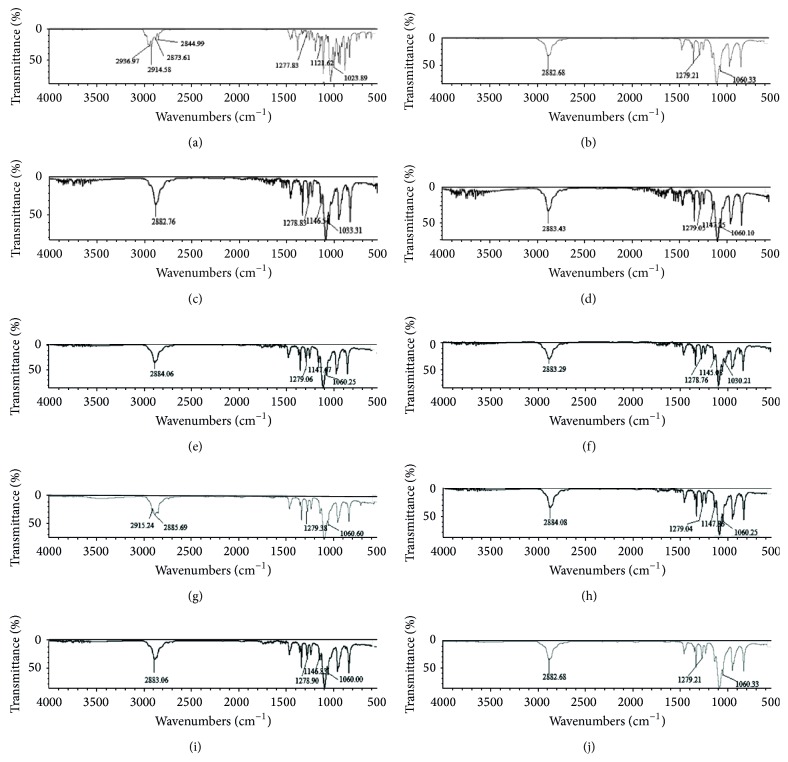
ATR-FTIR spectra of pure ARTM (a), PEG6000 (b), physical mixture of group-1 (c), freeze-dried mixture of group-1 (d), Cremophor-A25 (e), physical mixture of group-2 (f), freeze-dried mixture of group-2 (g), Poloxamer 188 (h), physical mixture of group-3 (i), and freeze-dried mixture of group-3 (j).

ATR-FTIR spectra of artemether (ARTM) indicated the presence of four characteristic peaks of C–H stretching vibrations at 2844.99 cm^−1^, 2873.61 cm^−1^, 2914.58 cm^−1^, and 2936.97 cm^−1^, C–O–O–C bending vibrations at 1121.62 cm^−1^, C–O–C stretching vibrations at 1023.89 cm^−1^ and 1277.83 cm^−1^, and C–H bending vibrations at 1451.05 cm^−1^ (Figure 3(a)).

The ATR-FTIR spectra of PEG6000 showed characteristic bands of C–H stretching vibrations at 2882 cm^−1^, O–H bending vibrations at 1341.02 cm^−1^ and 359.52 cm^−1^, C–O stretching vibrations at 1059.97 cm^−1^ and 1278.91 cm^−1^, and C–H bending vibrations at 1466.38 cm^−1^ (Figure 3(b)).

Physical and freeze-dried mixtures of group-1 showed characteristic bands of C–H stretching vibrations in functional group region at 2882.76 cm^−1^ and 2883.43 cm^−1^ which was single broader peak instead of four peaks of ARTM alone; in the fingerprint region, C–O–O–C bending vibrations of both physical and freeze-dried mixtures of group-1 were unaltered. C–O–C stretching vibrations of physical mixtures of group-1 were red shifted at 1033.31 cm^−1^ and 1278.83 cm^−1^, while its freeze-dried mixtures were also red shifted at 1060.12 cm^−1^ and 1279.05 cm^−1^, respectively. C–H bending vibrations of physical mixtures of group-1 were red shifted at 1456.79 cm^−1^ and 1465.91 cm^−1^, while its freeze-dried mixtures were red shifted at 1456.91 cm^−1^ and 1465.74 cm^−1^, respectively. ATR-FTIR spectra tell about presence and absence of bonding interaction among ARTM and excipients due to mixing, grinding, and freeze drying (Figures 3(c) and 3(d)).

ATR-FTIR spectra of Cremophor-A25 showed characteristic bands of C–H stretching vibrations at 2885.69 cm^−1^ and 2915.24 cm^−1^, O–H bending vibrations at 1341.58 cm^−1^ and 1359.54 cm^−1^, and C–O–C stretching vibrations at 1060.60 cm^−1^ and 1279.38 cm^−1^. In group-2 of SESDs of ARTM, physical and freeze-dried mixture showed characteristic bands of C–H stretching vibrations in the functional group region at 2883.29 cm^−1^ and 2884.06 cm^−1^, respectively, which indicated that C–H stretching vibrations of ARTM were masked as compared to pure ARTM and the C–H stretching vibrations of both physical and freeze-dried mixtures showed characteristics bands of Cremophor-A25. Similarly, in the fingerprint region, the C–O–O–C bending vibrations were unaltered which showed that there was no change in trioxane ring that indicated that our SESDs retained their antimalarial activity. The C–O–C stretching vibrations of physical mixtures of group-2 were red shifted at 1030.21 cm^−1^ and 1278.76 cm^−1^, while its freeze-dried mixtures were red shifted at 1060.25 cm^−1^ and 1279.06 cm^−1^; C–H bending vibrations of physical mixture of group-2 were red shifted at 1465.92 cm^−1^ and its freeze-dried mixture was red shifted at 1466.15 cm^−1^ (Figures 3(e)–3(g)).

ATR-FTIR spectra of Poloxamer 188 showed characteristic bands of C–H stretching vibrations at 2882.68 cm^−1^, O–H bending vibrations at 1341.58 cm^−1^ and 1359.38 cm^−1^, and C–O–C stretching vibrations at 1060.33 cm^−1^ and 1279.21 cm^−1^. When Poloxamer 188 was substituted with Cremophor-A25 in group-3 of SESDs of ARTM, the peak intensities and frequency of transmittance were not changed significantly. Physical and freeze-dried mixtures of group-3 showed characteristic bands of C–H stretching vibrations at 2883.06 cm^−1^ and 2884.08 cm^−1^ which indicated that C–H stretching vibrations of ARTM were masked and the C–H stretching vibrations of both physical and freeze-dried mixtures showed characteristics of Poloxamer 188. There was no change in C–O–O–C bending vibrations in physical and freeze-dried mixtures of group-3. C–O–C stretching vibrations of physical mixtures of group-3 were also red shifted at 1060 cm^−1^ and 1278.90 cm^−1^, whereas its freeze-dried mixture was red shifted at 1060.25 cm^−1^ and 1279.04 cm^−1^, respectively. C–H bending vibrations of physical and freeze-dried mixtures of group-3 were red shifted at 1466.13 cm^−1^ and 1466.11 cm^−1^, respectively (Figures 3(h)–3(j)).

The disruption in crystalline structure was similar to that of DHA [14]. The shifting and broadening agreed with previous ketoconazole results [15]. The shifting in the carbonyl stretching confirms a chemical interaction between ARTM and PEG, as occurs for norfloxacin [16]. Most bands were broad compared to pure ARTM, confirming an interaction between ARTM and PEG [17].

### 3.3. Differential Scanning Calorimetry

**Figure 4:**
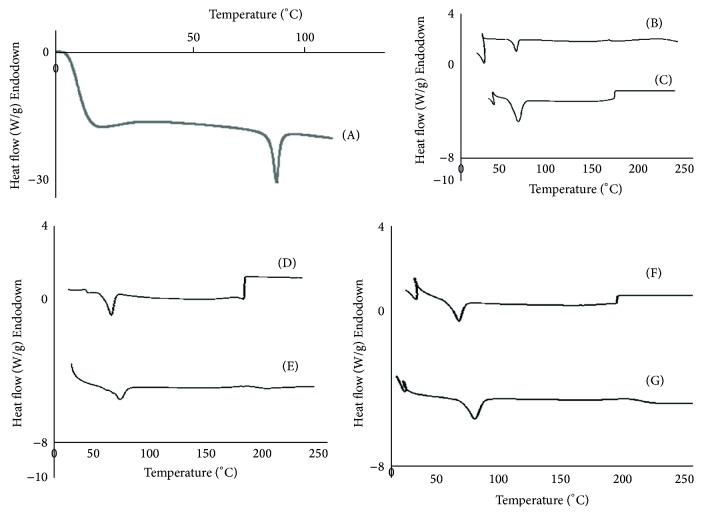
DSC thermogram of ARTM (A), physical mixture of group-1 (B), freeze-dried mixture of group-1 (C), physical mixture of group-2 (D), freeze-dried mixture of group-2 (E), physical mixture of group-3 (F), and freeze-dried mixture of group-3 (G).

The DSC thermogram of ARTM showed typical characteristics of a crystalline substance having one endothermic peak at 86.64°C while melting onset temperature at 84.86°C. An enthalpy change (Δ*H*) of ARTM was 56.68 J/g. The DSC thermogram of ARTM is shown in Figure 4(A).

Physical mixture of group-1 showed melting onset at 65.90°C, peak temperature at 66.55°C, and enthalpy change of 186.5 J/g, while its freeze-dried mixture showed decreased melting onset at 56.84°C, peak temperature at 61.90°C, and Δ*H* at 162.8 J/g. Both physical and freeze-dried mixtures of group-1 showed decreased melting peak temperature and increased enthalpy change. It was noted that decrease in melting endotherm [18] was more pronounced in case of freeze-dried mixture and increase in Δ*H* was more in case of physical mixture. All these changes were due to less crystalline nature of ARTM in SESDs of group-1 (Figures 4(B) and 4(C)).

Physical mixture of group-2 showed melting onset temperature at 61.84°C, peak temperature at 66.31°C, and Δ*H* at 109.4 J/g, while freeze-dried mixture of group-2 showed melting onset at 53.36°C, peak temperature at 60.93°C, and Δ*H* at 150.1 J/g. Both physical and freeze-dried mixtures showed lower endothermic peak temperature [18] and increased enthalpy change as compared to ARTM alone. The increase in enthalpy lowered melting endotherm effect which was more pronounced in case of freeze-dried mixture of group-2 than its physical mixture. Group-2 SESDs of ARTM were more stable than group-1 SESDs (Figures 4(D) and 4(E)).

### 3.4. Scanning Electron Microscopy (SEM) Studies

**Figure 5:**
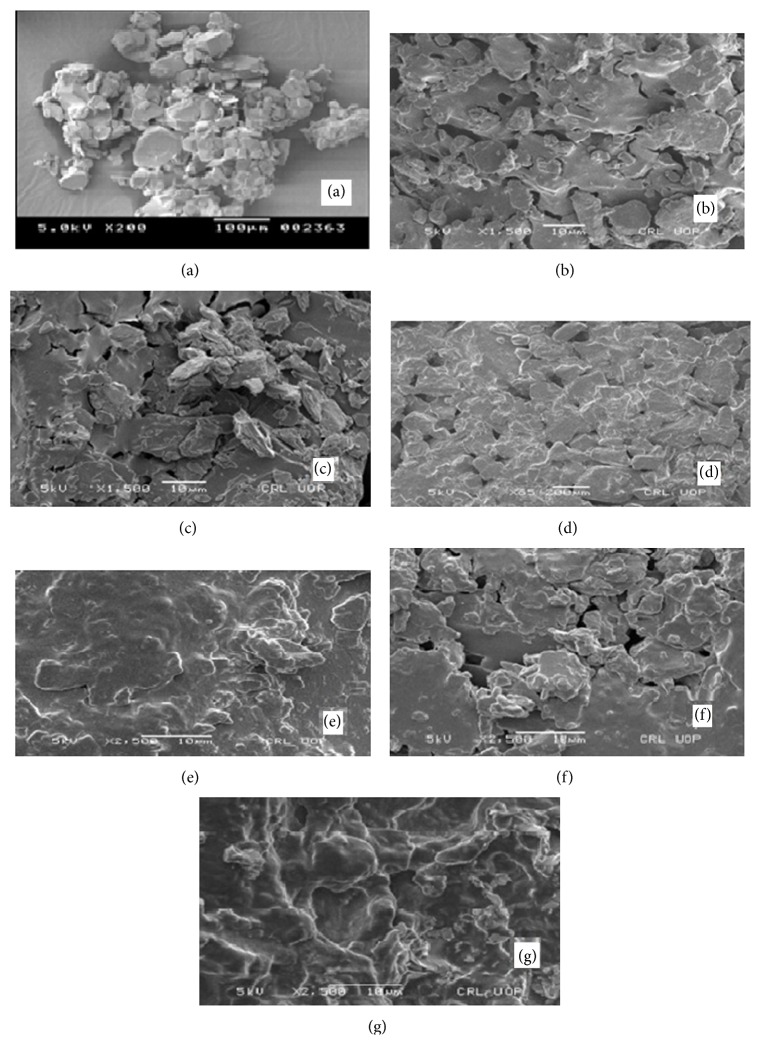
SEM of ARTM (a), physical mixture of group-1 (b), freeze-dried mixture of group-1 (c), physical mixture of group-2 (d), freeze-dried mixture of group-2 (e), physical mixture of group-3 (f), and freeze-dried mixture of group-3 (g).

Scanning electron microscopic photographs of artemether (ARTM) alone showed typical crystalline blocks of ARTM, while in group-1 SDs of ARTM, SEM showed that these crystalline structures of ARTM were decreased in size enormously having no sharp edges in both physical and freeze-dried mixtures of group-1. Scanning electron micrographs of physical mixture of group-2 showed formation of flakes representing amorphous agglomerates with smooth surfaces, whereas its freeze-dried mixture showed glassy appearance in addition to size reduction and embedment. SEM of physical mixture of group-3 in which Cremophor-A25 was substituted with Poloxamer 188, showed flakes having no smooth surface, while its freeze-dried mixture showed modified irregular shaped glassy appearance (Figure 5), comparable to the result of ARTM as obtained earlier [19].

### 3.5. Equilibrium Solubility Studies

**Figure 6:**
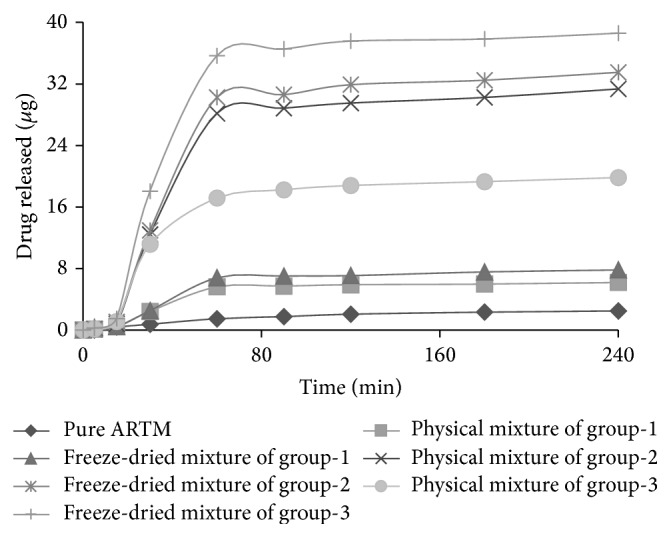
Dissolution profiles of ARTM alone and its formulations.

In group-1 of the prepared samples of artemether (ARTM), the solubility of physical mixture (PM) was increased up to 9 times (2.74 mg/mL) and solubility of its freeze-dried (FD) mixture was improved up to 15 times (4.74 mg/mL) as compared to ARTM alone (0.30 mg/mL). While in group-2 of SESDs, the solubility of physical mixture was increased up to 94 times (28.38 mg/mL) and its freeze-dried mixture was improved up to 121 times (36.33 mg/mL). In group-3 of SESDs when Cremophor-A25 was replaced with Poloxamer 188, the solubility of physical mixture was up to 65 times (19.49 mg/mL), while solubility of its freeze-dried mixture was increased further up to 135 times (40.56 mg/mL). In all cases, the solubility was in the decreasing order of FD > PM > ARTM (Figure 6).

All the physical and freeze-dried mixtures of all samples showed a substantial increase in equilibrium solubility. The increase in solubility was due to amorphous nature of prepared samples or inhibition of crystallization by polymers as obtained earlier [20–22]. Moreover, this increase in solubility can be a result of the formation of more soluble dispersion between the drug and the polymer [23]. The effect of temperature on solubility was similar to artemisinin [24], aspirin, and paracetamol [25]. Generally, solubility profile of SESDs of ARTM was agreed with data of XRD, FTIR, DSC, and SEM, which indicated that both procedures such as physical mixture method and freeze-dried method improved the solubility profile of ARTM.

### 3.6. Preparation and Characterization of Tablets

The quality control parameters of all prepared tablets were in accordance with official requirements [22]. Weight variation and friability were ±4.92% and <0.5%, respectively. The ARTM contents (%) in all the tablets ranged between 99.91 ± 0.73% and 102.01 ± 0.32%.

### 3.7. Dissolution Studies

From dissolution data (Table 1), it is found that rate of drug dissolution (*K*) is faster in all formulations as compared to pure ARTM as evident from zero order, first order, and Higuchian model analysis. Moreover, that rate of drug dissolution is faster in all products formulated by freeze drying compared to that of physical mixing. In addition, drug release data was best fit to the Higuchian model which illustrates that drug release from the products occurs through the diffusion process. This mode of drug release is further supported by *n*-value, that is, in range of 0.437–0.483. If *n* is equal to or less than 0.5, dissolution data follows the Fickian diffusion. The diffusion is Fickian when liquid diffusion takes place at slower rate than the rate of relaxation of polymeric chains. The *n*-value is assessed from the slope of Korsmeyer-Peppas curve [8]. Figure 6 shows dissolution profiles of ARTM alone and its formulations.

The rate of dissolution was increased by using Cremophor-A25 as well as Poloxamer 188 in addition to PEG6000, olive oil, Transcutol, and HPMC. By comparing physical and freeze-dried mixtures of SESDs of ARTM, the freeze-dried mixtures showed enhanced dissolution as compared to physical mixtures and ARTM alone. This enhanced dissolution of SESDs of ARTM was due to less crystalline structure and their conversion into amorphous form [18]. The order of decrease in dissolution was FD > PM > ARTM alone. The dissolution profile of SESDs of ARTM agreed with data of XRD, FTIR, DSC, and SEM, which indicated that both procedures such as physical mixture method and freeze-dried method improved the dissolution profile of ARTM. This increase in dissolution was comparable to that observed earlier for ARTM [26].

### 3.8. Stability Studies

**Figure 7:**
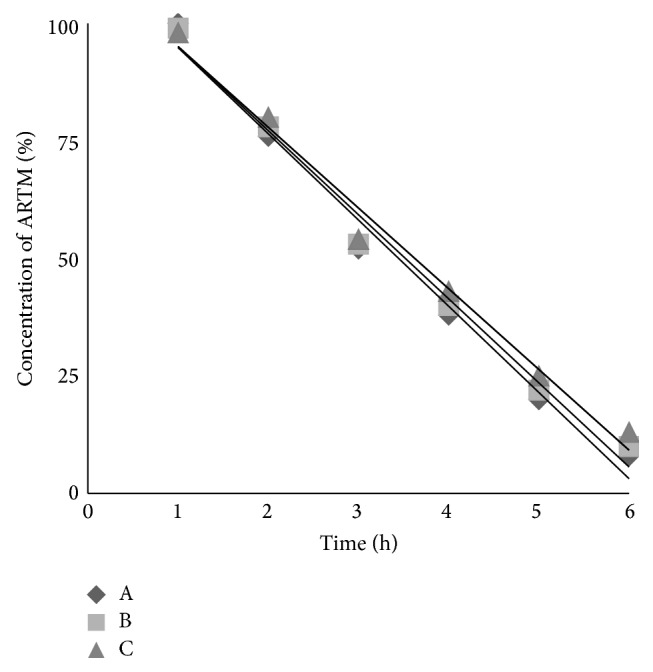
The changes of ARTM concentration percentage as a function of time in Hank's balanced salt solution (pH 7.4) at 37°C for ARTM alone (A), physical mixture of group-1 (B), and freeze-dried mixture of group-1 (C).

**Figure 8:**
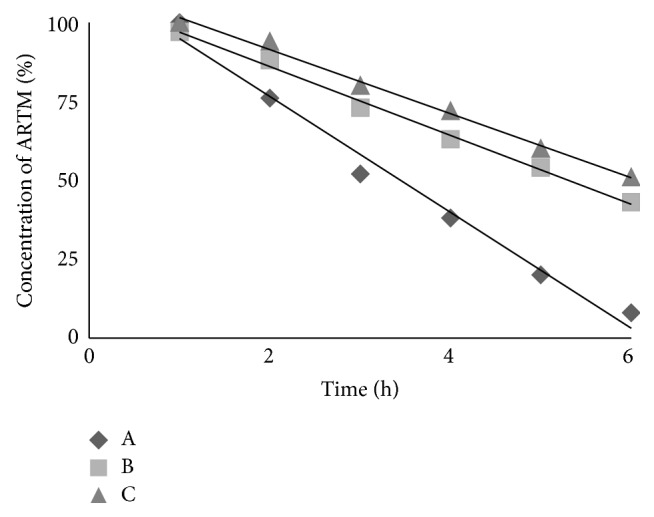
The changes of ARTM concentration percentage as a function of time in Hank's balanced salt solution (pH 7.4) at 37°C for ARTM alone (A), physical mixture of group-2 (B), and freeze-dried mixture of group-2 (C).

**Figure 9:**
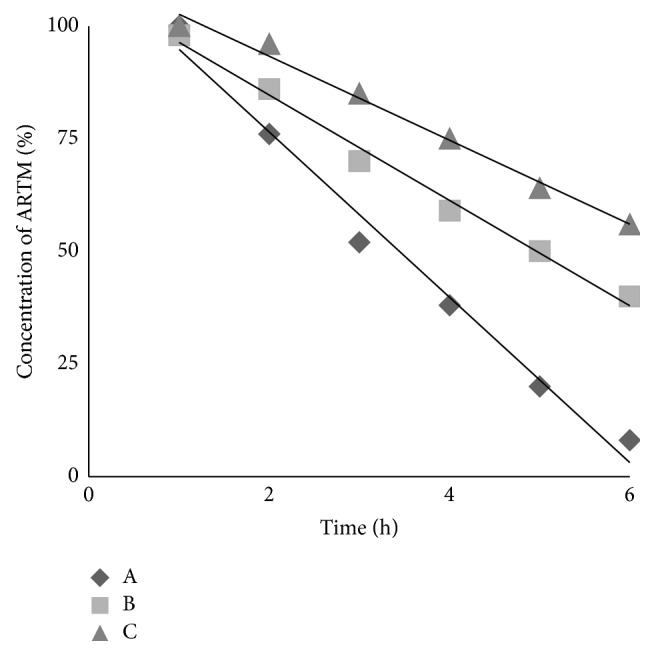
The changes of ARTM concentration percentage as a function of time in Hank's balanced salt solution (pH 7.4) at 37°C for ARTM alone (A), physical mixture of group-3 (B), and freeze-dried mixture of group-3 (C).

The stability of artemether (ARTM) in Hank's balanced salt solution of pH 7.4 was very poor and only 8% of ARTM was left at the end of 6 hours. Therefore, Hank's balanced salt solution pH 7.4 was chosen as medium for the stability analysis of ARTM in the SDs and SESDs at 37°C, as used previously for dihydroartemisinin [27]. The degradation of ARTM in Hank's balanced salt solutions (pH 7.4) followed first order reaction described by the following equation with *R* ^2^ > 0.984. The degradation rate constant values were calculated by linear regression of ln[*C*] and *t*. The changes of concentration of ARTM as well as SESDs of ARTM as a function of time were shown in Figures 7, 8, and 9.

The degradation rate constant (*k*) of ARTM alone was 0.52 h^−1^. Physical mixture (PM) as well as freeze-dried (FD) mixture of group-1 showed values of *k* 0.46 h^−1^ and 0.42 h^−1^, respectively. Physical and freeze-dried mixtures of group-2 of ARTM showed decreased *k* values 0.22 h^−1^ and 0.18 h^−1^, respectively. Similarly, physical and freeze-dried mixture of group-3 showed lowest values of *k* 0.25 h^−1^ and 0.11 h^−1^, respectively.

The rank order of the *k* values was ARTM alone > FD mixture > PM. The stability of ARTM in Hank's balanced salt solution at pH 7.4 was substantially improved by using PEG6000, Cremophor-A25, and Poloxamer 188, comparable to DHA [27, 28].

## 4. Conclusions

It can be concluded from our results that solubility and dissolution profile of artemether (ARTM) can be increased by preparing their self-emulsified solid dispersions (SESDs) with PEG6000, Poloxamer188, Cremophor-A25, olive oil, HPMC, and Transcutol by using freeze-dried method. The increase in solubility and dissolution profile of SESDs of ARTM agreed with data of XRD, FTIR, DSC, and SEM, which indicated that self-emulsified solid dispersions by freeze-dried method improved the physicochemical properties of ARTM.

## Figures and Tables

**Table 1:** Kinetic analysis of dissolution data.

Formulations	Zero order model		First order model		Higuchi model		Korsmeyer-Peppas model
	*K*	*R* ^2^	*K*	*R* ^2^	*K*	*R* ^2^	*n*
Pure ARTM	0.013	0.7857	0.000	0.7913	0.171	0.9569	0.549
Physical mixture of group-1	0.036	0.5417	0.000	0.5622	0.478	0.8383	0.459
Freeze-dried mixture of group-1	0.044	0.5961	0.000	0.6199	0.589	0.8535	0.483
Physical mixture of group-2	0.181	0.5622	0.002	0.6719	2.403	0.8322	0.473
Freeze-dried mixture of group-2	0.194	0.5651	0.003	0.6839	2.573	0.8336	0.475
Physical mixture of group-3	0.115	0.5002	0.001	0.5739	1.546	0.8346	0.437
Freeze-dried mixture of group-3	0.227	0.5121	0.003	0.6677	3.035	0.8215	0.450
